# Exploring the impact of endometriosis on women’s lives: A qualitative study in Iran

**DOI:** 10.1002/nop2.744

**Published:** 2020-12-22

**Authors:** Masoumeh Namazi, Zahra Behboodi Moghadam, Armin Zareiyan, Mina Jafarabadi

**Affiliations:** ^1^ School of Nursing & Midwifery Tehran University of Medical Sciences Tehran Iran; ^2^ Public Health Department Nursing Faculty Aja University of Medical Sciences Tehran Iran; ^3^ Reproductive Health Research Center Tehran University of Medical Sciences Tehran Iran

**Keywords:** endometriosis, health, Iranian women, life experience, nurses, nursing, qualitative study

## Abstract

**Aim:**

Considering the wide impact of endometriosis on various aspects of health, this study aimed to explore the impact of endometriosis on Iranian women's lives.

**Design:**

The present study used a qualitative approach and conventional content analysis.

**Methods:**

Twenty patients suffering from endometriosis referring to Imam Khomeini Hospital in Tehran took part in this study. The sampling was done purposefully until data saturation. After deep semi‐structured interviews, the content analysis of the interviews was done according to the steps proposed by Zhang and Wildemuth.

**Results:**

The mean age of the participants was 34.53 (*SD*: 5.81) years (range: 23–43) with duration of illness of 6.30 ± 5.86 years. Their educational level varied from high school to university, and most of them were employed. Analysis of the data from participants' experiences led to the formation of 5 categories under the titles "Physical suffering, instability of marital life, mental disorder, disruption in social life and self‐care" and 11 subcategories.

**Conclusion:**

The findings of the present study showed that endometriosis can adversely affect women's lives including physical, sexual, psychological and social problems. Although in some cases patients adopt different strategies for self‐care, the need for more support is felt.

## 1 INTRODUCTION

Endometriosis is a chronic disease that has been less diagnosed and is defined as implantation of endometrial stromal implants outside the uterine cavity (Burkman, 2012; Kiykac Altinbas et al., 2015; Moradi et al., 2014). It is estimated that approximately 5%–10% of women of reproductive age (176 million people in the world) are afflicted with the disease (Deguara et al., 2012; Harada et al., 2016; Nnoaham et al., 2011). The economic burden of endometriosis is very high and is similar to other chronic diseases such as diabetes and Crohn's disease ( Simoens et al., 2012).

Despite the high morbidity of endometriosis and the high cost of its health care, the precise aetiology of the disease is still unclear and only a few risk factors have been identified (Missmer et al., 2010). The most common symptom in women with endometriosis is pelvic pain and infertility (Montanari et al., 2013). Pain can occur in a variety of forms including menstrual pain (dysmenorrhoea), pain during intercourse (dyspareunia), chronic pelvic pain and cyclic dysuria (Moradi et al., 2014). The definitive diagnosis of endometriosis is possible only through laparoscopy and histological confirmation. There is currently no definitive treatment for endometriosis, and therapeutic and surgical treatments are only used to relieve the symptoms (Hjordt Hansen et al., 2014; Young et al., 2016).

## 2 BACKGROUND

Endometriosis can significantly affect various aspects of physical, mental and social health of women (Hjordt Hansen et al., 2014). According to previous studies, women with endometriosis are subject to complications such as infertility, chronic pelvic pain, anxiety, depression, mood swings, decreased quality of life, sexual dysfunction and marital problems (Barbara et al., 2017). Studies in Iran have also shown that endometriosis is associated with major difficulties such as pain, loss of psychosocial function and a significant decrease in patients’ quality of life (Riazi et al., 2014). Unfortunately, notwithstanding that life with endometriosis is very difficult for many patients, the problems of these patients have not received attention and women suffer from the harmful effects of the disease for a long time. Research shows that delay in diagnosis is a major problem in the disease. The median duration of diagnosis from the onset of pain is about 8 years in England and 12 years in the United States (Moradi et al., 2014). Therefore, people with this disease spend a significant part of their lives involved with the disease and its impact on their various aspects of health. Unfortunately, so far, little qualitative studies have been done on this disease and most studies have been conducted quantitatively in Western countries. In addition, the existence of methodological limitations in many studies and significant gaps in resources highlights the need for further studies in different societies. On the other hand, the experience of patients with endometriosis is influenced by the socio‐cultural context and varies from one country to another and the role of culture is not negligible. Considering the above, the present study aimed to explore the impact of endometriosis on Iranian women's lives.

### 2.1 Design

The present study was conducted from December 2017–July 2018 using qualitative approach and conventional content analysis. Conventional content analysis is generally used with a study design whose aim is to describe a phenomenon when existing theory or research literature about it is limited. Researchers avoid using pre‐conceived categories, instead allowing the categories and names for categories to flow from the data.

## 3 METHODS

Twenty patients with endometriosis participated in this study. The target population was women with endometriosis referred to Imam Khomeini Hospital in Tehran. The purposive sampling method was used to recruit the participants, and the sampling was continued until data saturation. The inclusion criteria for the study included marriage, being at the reproductive age (15–45 years) and definitive diagnosis of the disease through laparoscopy. The exclusion criteria were unwillingness to participate in the study and other causes of pelvic pain. In this study, the data were saturated with 17 people, but we also interviewed 3 others for more certainty. Written and informed consent for participation in the research was obtained from the participants. They were assured that they could withdraw from the research whenever they wished, and all their information would remain confidential. Data collection was done through individual in‐depth semi‐structured interviews. The face‐to‐face interviews were held in a dedicated room, and each lasted between 45–90 min. Interviews started with questions such as "What is your experience of life with endometriosis?" and "What effect did endometriosis have on your life?" and continued with exploratory questions such as "What do you mean?", "How?", "Why?" and "You may explain more about this." Interviews were recorded with the permission of the patients, and on the same day, each interview was written word‐for‐word and then coded, and after the analysis of each interview, the next interview was carried out.

To determine the rigour of the qualitative data, Lincoln and Guba's criteria were used, including credibility, dependability, conformability, transferability and authenticity of the data (Polit & Beck, 2008). To validate data collection, semi‐structured deep interviews and continuous involvement of the researcher in the data and continuous comparison were used. To confirm the authenticity of the data, the extracted codes were returned to the interviewer to confirm or correct them. Dependability was obtained through a review by members of the research team and those who were qualified. Data transferability was provided through a complete presentation of the research methodology, along with providing examples of participants' statements, to allow others to follow the research path.

### 3.1 Analysis

The content analysis of interviews was done according to the steps proposed by Zhang and Wildemuth, which included:

Preparing data (implementing and writing interviews): In this stage, the recorded interviews were converted to text format. All interviews were transcripted to reveal a clear model of the thoughts, behaviours, ideas and experiences of the study participants.

- Defining and categorizing data analysis units: Each interviewed text was entered into qualitative data analysis software as a unit of analysis. Before coding, the entire text of the interview was read several times so that the researcher was fully acquainted with the data. They were then coded by specifying meaningful units.
- Specifying categories and patterns of data coding: At this stage, a pattern was designed to develop categories and subcategories. Categories were extracted from codes inductively. The codes were first placed in subcategories due to similarity, and then, the subcategories formed a category based on their relationship to each other. The categories were organized in such a way that they had internal compatibility and external incompatibility.
- Evaluation of the sample coding pattern: For this purpose, the researcher coded a sample of text to control the consistency of the coding by two members of the research team. Discrepancies in the rules regarding coding or classification of codes were resolved through discussions between the research teams.
- Coding the full text of the interview: After the researcher and two members of the research team agreed on coding stability, a reproducible process was achieved and the coding process was extended to the whole text. During the coding process, the researcher continuously monitored the coding to ensure that there was agreement between the extracted codes based on the researcher's inference and the views of the study participants and the research team.
- Evaluating the coding stability: After full‐text encoding, the coding stability was checked again. During the analysis process, the researcher controlled the consistency of the coding, including the primary codes, their placement in the subcategories and the formation of categories with other people, including two members of the research team and experts in qualitative research.
- Extracting the results from the coded data and reporting them: At this stage, the characteristics and dimensions of the categories were discovered and the relationships between them were identified (Zhang & Wildemuth, 2009).

The MAXQDA software (v. 10, VERBI Software GmbH, Berlin) was used for data analysis.

### 3.2 Ethics

Sampling was done after approval from the research council of the Faculty of Nursing and Midwifery of Tehran University of Medical Sciences and obtaining permission from the Ethics Committee of this university (Approval Number: IR. TUMS.FNM.REC.1396.4242).

## 4 RESULTS

Participants included 20 married patients with endometriosis who were diagnosed with laparoscopy. The age of the participants was between 23–43 years, and the mean age was 34.53 ± 5.81 years. Other demographic characteristics of the participants in the study are presented in Table 1. Analysis of data from participants' experiences led to the formation of five categories and 11 subcategories. Table 2 shows the categories and subcategories of the research.

**TABLE 1:** Demographic characteristics of the participants

Demographic characteristics	*N* (%) or mean (*SD*)
Education	
High school	4 (26.66)
Diploma	5 (33.33)
University	11 (55)
Employment status	
Unemployed	9 (60)
Employed	11 (55)
Number of children	
0	16 (80)
1	2 (13.33)
2	2 (13.33)
The economic situation	
Low	3 (20)
Moderate	14 (70)
High	3 (20)
Husband's education	
High school	2 (13.33)
Diploma	5 (33.33)
University	13 (65)
Duration of illness (years)	6.30 ± 5.86
Duration of marriage (years)	9.76 ± 6.12
Age at diagnosis (years)	24.65 ± 6.58
Interview time (min)	55.66 ± 13.87

**TABLE 2:** Categories and subcategories resulting from the research

Categories	Subcategories
Physical suffering	Menstrual disorders
	Disabling pelvic pain
Instability of marital life	Emotional tension with the spouse
	Sexual dissatisfaction
Psychological disorder	Frustration
	Self‐condemnation
	Feeling of repeated failure
Disruption in social life	Isolationism
	Disruption in daily activities
Self‐care	Lifestyle change
	Pain management

### 4.1 Category 1. Physical suffering

This category includes two subcategories of menstrual disorders and disabling pelvic pain.

1–1. Menstrual disorders: Patients suffered from severe bleeding and clotting, spotting before and after menstruation and irregular menstruation. Severe bleeding and clotting were reported by most participants. For example, patients expressed the following:

My hemorrhage is very severe during my period, I can't even move dithe bathroom repeatedly (Participant No. 4).

Spotting before and after menstruation was one of the other common problems raised by the participants in the research:

Two days before the period, I see brown spots and it last until after of menstruation. For example, if my period lasts for 7 days, then I have a spotting for seven days after it. (Participant No. 7)

The irregular menstruation was also another complaint raised by the participants:

My periods are very irregular. Each month, a few days back or forth. y periods are not predictable at all (Participant No. 2)

1–2. Disabling pelvic pain: Patients suffered from severe pain in the waist and abdomen, pain in the ovaries and vague pelvic pain:

During the period under my belly was very painful, pain was very severe and not tolerable at all (Participant No. 15)

Another participant said:

I feel my ovary is exploding, I can't do anything, I just cry (Participant No. 3)

My pain starts from the middle of my stomach, then it spreads around my abdomen and my lower back and legs. (Participant No. 12)

Most people described the pain as unbearable:

My pain was so terrible, I did not like to be alive, I was very annoyed, I had to go to the hospital when I had pain, It was so hard to breathe, I wanted to die (Participant No. 5)

The severity of these pains in some people was not resolved by painkiller:

I was hospitalized for the very pain that I had at the time of the period and I did not get rid of any pain, so that I had to take morphine. (Participant No. 20)

### 4.2 Category 2. Instability of marital life

This category consisted of two subcategories of emotional tension with the spouse and sexual dissatisfaction. 2–1. Emotional tension with the spouse: Some participants suffered from the lack of husband support during the course of the illness:

When your husband does not support you and most importantly, he does not pay for the treatment, it's very difficult. The problem is, on the one hand and these collisions on the other side (Participant No. 17)

Some people also blamed themselves and, therefore, tended to be separated from their husbands:

I constantly say to my husband that we should be separated and I feel that my husband is so annoyed because of my illness. After divorce, my husband gets comfortable. (Participant No. 16)

2–2. Sexual dissatisfaction: One of the most common problems is pain during intercourse: *"During my intercourse, I have a lot of pain that I can't bear at all, this pain lasts even a few days" (Participant No. 6),* and also decreased libido, inability to achieve orgasm and reduction in the number of intercourses from other sexual problems mentioned by the participants in the study:

It's so annoying that I have no desire for intercourse at all. (Participant No. 14)

During intercourse, I have pain so much that I never enjoy it (Participant No. 2)

I've spotted before and after the period for a few days and therefore I can't have intercourse. The number of my intercourses has dropped, which makes me very annoyed (Participant No. 7)

### 4.3 Category 3. Psychological disorder

This category consisted of three subcategories: frustration, self‐ condemn and feeling of repeated failure.

3–1. Frustration: Frequent recurrence of illness and lack of response to medical and surgical treatments to reduce the complications of the disease has been a disappointment in some patients:

My illness recurs repeatedly. I was looking for treatment, but the treatment did not work. I was disappointed with the treatment of the disease (Participant No. 5)

Some people have also been disappointed with having a child:

I tried all the techniques of fertility, but it did not work. I'm disappointed with having a child. (Participant No. 19)

3–2. Self‐ condemnation: Some people suggested that many complications of the illness made them compare themselves with others:

I compare myself with others, seeing others easily getting pregnant, not having problems. I compare myself with them. (Participant No. 14)

The suffering of disease‐induced infertility has led some to consider themselves different from other people:

Sometimes I think I have weakness due to endometriosis and I can't be a mother, so I feel I’m different from others. (Participant No. 12)

3–3. Feeling of repeated failure: Feeling of failure in patients with endometriosis can be due to various causes such as recurrence of illness, infertility and failure of assisted reproductive technologies (ART). Participants stated:

I have undergone surgery for several times, but my illness is recurring. (Participant No. 9)

I got married three years ago and did not prevent pregnancy at all but did not get pregnant, the doctor told me that the cause of my infertility is the adhesion of the uterine tubes (Participant No. 17)

I've done hysteroscopy twice, IUI several times and IVF twice, but none of them was successful (Participant No. 18)

### 4.4 Category 4. Disruption in social life

This category consisted of two subcategories: isolationism and disruption in daily activities.

4–1. Isolationism: Patients stated that due to lack of understanding of the illness by relatives and colleagues they prefer to be alone:

My husband's family does not understand me at all. My mother‐in law always asks me when I want to have children. So, I would prefer not to see them (Participant No. 2).

At work, my colleagues do not understand my condition at all. I have to take leave to follow up treatment, but in most cases, my chairman disagrees with the leave request (Participant No. 1).

4–2. Disruption in daily activities: The severity of dysmenorrhoea was, in some people, so much that it caused their inability to do the routine activities: *"When I have pain, I can't do anything at all. My husband does the homework " (Participant No. 2)*. The severity of bleeding and pain in some people was such that the need to rest at home was unavoidable for them: *"My bleeding and pain during Menstruation is so much, which I can‘t go to work at all." (Participant No. 18)*.

### 4.5 Category 5. Self‐care

This category consisted of two subcategories: lifestyle change and pain management.

5–1. Lifestyle change: Participants in the study had adopted different ways of changing lifestyles. Some suggested that getting information about the disease and its nature made it less stressful:

I search the net about the illness and its complications. When I have more information about the disease, I feel less stressful. (Participant No. 6)

Some were also entertaining themselves with the job: *When I'm doing work, I do not even have the opportunity to think about the disease. (Participant No. 13)*.

Exercising and walking, as well as travelling with friends, constitute other ways of self‐care in these patients:

Every day I go to the park, exercise and walk. If I stay at home I blame myself. So, I try to do something. (Participant No. 9)

I have a few friends who are very close and we go out together. So, I think less about my disease. (Participant No. 20)

5–2. Pain management: Patients used different ways to reduce pain. For example, some have turned to traditional medicine:

My friend introduced me to a doctor of traditional medicine. He gave me a series of herbal remedies, since then, I feel less pain. (Participant No. 3)

Water therapy and heat therapy were also other methods of managing pain in patients:

During the period I like to use water therapy, I like to get warm water on my skin. I feel my pain subsides (Participant No. 5)

When I'm in pain, I put a warm towel on my waist and my abdomen. (Participant No. 19)

## 5 DISCUSSION

The present study aimed to explore the impact of endometriosis on Iranian women's lives. The results showed that endometriosis can adversely affect women's lives including physical suffering, instability of marital life, psychological disorder and disruption in social life. Physically, endometriosis causes menstrual disorder, including severe bleeding and clotting, spotting before and after menstruation and irregular menstruation. These results are in line with the findings of other studies (Ballard et al., 2006; Moradi et al., 2014; Riazi et al., 2014). Participants in the research stated that they have had many menstrual disorders even from adolescence, but they have refused to go to the doctor because they have considered it normal. So, delay in diagnosis is a common problem in patients, which can aggregate the physical complications of endometriosis. It seems that increasing the awareness of women and girls about normal menstruation and distinguishing it from abnormal conditions and creating sensitivities for referring to related centres in the event of any abnormal condition can partly reduce this problem (Riazi et al., 2014).

Disabling pelvic pain is another physical problem that most patients complain of, as other studies have mentioned (Marqui, 2015; Oehmke et al., 2009; Riazi et al., 2014). In the present study, patients suffered from pelvic retardation complaints and in some cases the pain was so severe and unbearable that individuals were admitted to the hospital for pain relief. Although the mechanism of pain associated with endometriosis is not well known, menstrual flow has been introduced as a stimulant for inflammatory pain by activating the immune system and peripheral nervous system terminals. Also, endometriosis can cause chronic pelvic pain caused by adhesion, inflammation or ovarian cyst (Riazi et al., 2014).

Instability of marital life is one of the most important problems of women with endometriosis that can occur in the forms of emotional tension with the spouse and sexual dissatisfaction. Endometriosis complications such as infertility, dyspareunia, depression and anxiety can be a threatening factor in the emotional intimacy between couples (Ameratunga et al., 2017). Some participants in the study were complaining about the lack of support from the spouse and the controversy and disagreement with him due to the illness. The results of other studies are also consistent with the present study (Ameratunga et al., 2017). One of the most important threats to the emotional intimacy of couples is sexual problems. According to Denny and Mann (2007), dyspareunia for some women was unbearable to the extent that it disrupted their intercourse with their partners. Also, according to some participants in this study, avoidance of sexual intercourse has led to an increase in tension and verbal conflict with partners (Denny & Mann, 2007). Deep dyspareunia is the most common complaint in sexually active women and is associated with adhesion, endometrium, uterine retroversion and endometriosis lesions located along the uterosacral ligaments (Deguara et al., 2012). In the present study, participants were suffering from multiple sexual problems, such as dyspareunia, loss of libido, inability to achieve orgasm and reduction in the number of intercourses due to spotting and dyspareunia. According to previous studies, dyspareunia induced by endometriosis can have negative effects on sexual satisfaction and sexual function of partners (Pluchino et al., 2016; Riazi et al., 2014; Tripoli et al., 2011). In the study by Kiykac Altinbas et al. (2015), 53.1% of people with endometriosis had a decreased libido although this did not reduce the number of sexual intercourses. Researchers consider this as a kind of defence mechanism in people with endometriosis. Also, the fear of breaking the relationship with the partner and separation causes unwanted sex (Kiykac Altinbas et al., 2015). According to a study by Barbara et al. (2017), endometriosis, especially in association with severe dyspareunia or chronic pelvic pain, can have negative effects on sexual performance of women. Types of endometriosis, which are associated with rectovaginal involvement and deep aggression, result in severe sexual dysfunction, which is due to the higher prevalence of dyspareunia in these people (Barbara et al., 2017).

Other effects of endometriosis include psychological problems. In the present study, participants suffered from psychological discomfort, such as frustration and a feeling of repeated failure. Many people were disappointed with treatment and were frustrated with having a child due to lack of response to medical and surgical treatments and frequent recurrence of disease. Problems and complications caused by the disease have caused some people in their subconscious to always compare themselves with others and blame themselves in some way. Also due to infertility, some people considered themselves different from others and considered themselves defective in the role of femininity and motherhood. These results are in line with the findings of other studies where most participants suffered from weakness, failure, anxiety, depression, frustration and lack of energy (Moradi et al., 2014; Riazi et al., 2014).

According to the results of this study, the social life of individuals can also be severely affected by the disease. Isolation due to the lack of understanding of the disease by the people or colleagues was a problem that was raised by some participants in the research. Due to the uncertainty about the exact aetiology of the disease, people with endometriosis suffer from distress and talking about the disease is difficult for them, which leads to the isolation of the individual and their being rejected from the community (Bitzer, 2011; Culley et al., 2013). According to Gilmour et al. (2008), participants in the study stated that they could not talk about their illness with male employers, which caused many problems in their work environment. Also, due to digestive problems and abdominal pain and the need for rest, they were not able to stay in appointment for a long time. Many people were not able to do full‐time work and even lost some jobs. Failure to participate in social activities, exercise, walking and party due to intestinal and bladder problems, fatigue and chronic pain were other problems (Gilmour et al., 2008). According to Moradi et al. (2014), most participants reported that due to mental changes caused by illness, pain, stress and anxiety, their relationship with others has been severely impaired in such a way that disputes and verbal controversy among them increased (Moradi et al., 2014). According to the findings from the present study, complications of the disease such as dysmenorrhoea and severe bleeding caused disturbances in daily activities such as inability to do homework and work, absence from the work and the need for rest at home. According to Fourquet et al. (2010), the participants in the study stated that endometriosis causes their disability in doing housework (79%), doing work (66%), participation in social activities (48%) and child care (45%) (Fourquet et al., 2010). In a study by Soliman et al. (2017), there was a direct correlation between the severity and number of endometriosis symptoms and the absence of the work and disability in doing work (Soliman et al., 2017).

Finally, patients used different strategies for self‐care. These strategies were divided into two groups: lifestyle change and pain management. Lifestyle change strategies include increasing information about the disease to reduce stress, being entertained by work, walk and exercise and being with friends. Management of pain also involves the use of traditional medicine to reduce pain, water therapy and heat therapy. In other studies, patients also used different non‐pharmacological methods to overcome the complications of the disease. For example, according to Gonçalves et al. (2017), yoga practice twice a week for 90 min in 8 weeks reduces chronic pain and improves the quality of life in endometriosis patients (Gonçalves et al., 2017). According to a study by Mira et al. (2015), treatment using TENS (transcutaneous electrical nerve stimulation) can improve deep dyspareunia and quality of life among endometriosis patients (Mira et al., 2015).

The findings of present study provide information about the various impacts of disease, so the health providers could make health policy decisions to improve services for patients. Further studies are also needed to explore the impact of endometriosis on partners of patients and the ways to overcome the adverse effects of disease.

As the main limitation, due to the nature of qualitative studies, our findings cannot be generalized. Also, because the staging of endometriosis according to the American Society for Reproductive Medicine (ASRM) classification is not done in Iran, we were unable to categorize patients in the present study. The main strength of the present study lays in exploring the impact of endometriosis on women's lives. However, this strength is undermined by the generalizability issue inherent to qualitative studies.

## 6 CONCLUSION

The findings of present study showed that endometriosis can adversely affect women's lives including physical, sexual, psychological and social problems. Although in some cases patients adopt different strategies for self‐care, the need for more support is felt.

## CONFLICT OF INTEREST

The authors declare that they have no competing interests.

## AUTHOR CONTRIBUTIONS

Masoumeh Namazi: Substantial contributions to conception and design of data; Armin Zareiyan and Mina Jafarabadi: Revision of the manuscript critically for important intellectual content; Zahra Behboodi Moghadam: Final approval of the version to be published. Zahra Behboodi Moghadam: Accountable for all aspects of the work in ensuring that questions related to the accuracy or integrity of any part of the work are appropriately investigated and resolved.

## Data Availability

No data are available online. All supporting data can be provided upon request to the authors.
